# Activating interactions of sulfanilamides with T cell receptors*

**DOI:** 10.4236/oji.2013.33019

**Published:** 2013-09

**Authors:** Stephan Watkins, Werner J. Pichler

**Affiliations:** 1 Department of Rheumatology, Clinical Immunology and Allergology, Inselspital/University Hospital of Bern, Bern, Switzerland; 2 Department of Graduate Cell Biology and Biomedical Sciences, University of Bern, Bern, Switzerland

**Keywords:** p-i Concept, Drug Hypersensitivity, Sulfamethoxazole, TCR Signaling, TCR Specificity, TCR-Modelling, T Cell Clones

## Abstract

Activation and expansion of drug reactive T cells are key features in drug hypersensitivity reactions. Drugs may interact directly with immune receptors such as the human leukocyte antigens (HLA) or the T-cell receptors (TCR) itself, the pharmacological interaction [p-i] concept. To analyze whether the drug sulfamethoxazole (SMX) interacts directly with the TCR and thereby contributing to signaling and T cell activation, we analyze two SMX specific T cell clones (TCC “1.3” and “H13”). Proliferation to SMX and 11 related sulfanilamides, Ca++ influx in drug stimulated T-cells and the inhibitory effect of non-reactive sulfanilamides on SMX stimulation were analyzed. *In silico* docking of SMX and related sulfanilamide to the TCR were used to identify possible drug binding sites, and correlated to *in vitro* data to find the correct docking. In Ca++ influx assays, reactions occurred as early as 14 sec after adding SMX to TCC and APC. The broadly reactive clone (“H13”) was stimulated by 5 additional sulfanilamide, while one TCC (“1.3”) was reactive exclusively with SMX but not other sulfanilamides. Competition experiments with sulfanilamide inhibited SMX induced Ca++ influx and proliferation of the TCC 1.3 in a dose dependent way. Docking experiments with SMX and related sulfanilamides confirmed and explained the *in vitro* data as docking localized binding sites for SMX and the 5 stimulating sulfanilamides on the CDR2*ß* domain of the clone H13, while the 6 non-stimulatory SA failed to bind. In TCC 1.3, SMX could be docked on the CDR3α of the TCR. The other, non-stimulatory but inhibitory SA could also be docked to the same site. The combined analysis of *in vitro* and *in silico* data show that sulfanilamide can bind directly to TCRs. It shows that TCR, like other receptors, appear to be reamenable to manipulations by small molecules.

## 1 Introduction

Sulfanilamides (SA) can elicit hypersensitivity reactions, resulting in various diseases [1,2]. A majority of these reactions involve the expansion of a few drug specific T-cells, which can coordinate an inflammatory immune response resulting in a clinical picture, which often involves the skin [3,4]. How the immune system is stimulated in drug hypersensitivity reactions is still a matter of debate. The hapten-concept postulates that a small molecular compound like a drug or a drug-metabolite is too small to be immunogenic per se. If the small chemical forms covalent bonds to a protein, the hapten-carrier complex is stable and remains coupled to the amino acid even after intracellular processing to small peptides. These hapten modified peptides are immonogenic when presented by the highly polymorphic proteins of the major histocompatibility complex (MHC), the so-called Human Leukocyte Antigens (HLA). The hapten/peptide-HLA complex can function as a neo-antigen for some T cells able to interact with it. Examples of haptens are penicillinG and other beta-lactam antibiotics, which via their *β*-lactam ring can bind directly to lysine or serine groups within proteins. Sulfamethoxazole (SMX) is an example for a pro-hapten, which is metabolized to a highly reactive SMX-NO intermediate. The latter binds as a hapten covalently to the sulfur of cysteins, however alone can also elicit immune responses [2,5]. Clinical effects of such reactions are heterogeneous, including anaphylaxis, haemolyticanaemia, or contact dermatitis, to name just as few [1].

A quite different, alternative explanation for immune stimulations by small molecular drugs leading to hyper-sensitivity reactions was recently developed by our group. It is termed “p-i-concept”, standing for “pharmacological interaction with immune receptors” [6,7]. Descriptively it postulates that a drug may interact directly with some of the highly variable immune receptors, in particular with some of the highly polymorphic HLA-molecules and TCR. This is akin to drug interactions with other receptors, where small molecular pharmaceuticals may bind to an enzymatic or ligand binding site. The p-i concept does not imply the formation of stable antigenic hapten-carrier determinants. Neither drug metabolism nor protein processing is required, as the drug itself, and not a hapten modified peptide, is binding to the receptors. All interactions involved are mediated by van der Waals forces, salt bridges, or other electrostatic interactions without any direct covalent changes. Such drug-protein interactions are common and are mostly without functional consequences. However, some drugs may fit into some of these immune receptors quite stably, giving rise to immune stimulations [4,5]. Indeed, the chance of functionally relevant drug bindings to some immune recaptors is probably related to the enormous polymorphism of specific immune receptors. One estimates that one individual harbors 10^9^ - 10^11^ different T cells and distinct TCR based on CDR3 polymorphism, offering an enormous amount of variable binding sites [8–10]. Also the polymorphism of major histocompatibility complex (MHC)-alleles within the population is large, with presently more than 9300 different alleles (http://www.ebi.ac.uk/imgt/hla/stats.html). The chance that some drugs may bind to a particular immune recaptor with a rather strong affinity is thus relatively high [10, 11].

One can differentiate between two forms of the p-i concept, and possibly more if other proteins of the immunological synapse are included [12]. The labile drug binding to immune receptors may be stimulatory for the immune system by altering either the HLA-peptide-complex (p-i HLA) or the TCR directly (p-i TCR). The p-i HLA concept has been well documented in HLA associated drug hypersensitivity reactions [13,14]. As an example, severe Carbamazepine hypersensitivity in Han Chinese occurs almost exclusively in HLA-B*15:02 positive individuals, which has been shown to bind Carbamazepine via non-covalent bonds [15,16]. Peptides eluted from the HLA were devoid of bound carbamazepine, arguing against a haptenized carrier molecule. Similarly, the drug Abacavir binds selectively to HLA-B*57:01, in particular to the F-pocket of this allele. It may then be recognized by T-cells, which are reactive to this modified MHC-molecule within 60 - 300 sec, clearly before Abacavir uptake, metabolism and processing could have taken place [12]. Interestingly, binding of Abacavir may alter the ability of HLA-B*57:01 to bind the usual peptides, and in the presence of Abacavir the MHC molecules select a modified peptide repertoire for presentation [17–19]. Thus, Abacavir may act as a peptide exchange molecule, much like invariant chains in HLA class II molecules, stabilizing an open or accepting form of the MHC [20]. The modified peptides presented may give rise to autoimmune or alloimmunereactions.

A number of previous findings support the concept that direct drug binding to TCR (p-i TCR) may also play a role in drug related T cell stimulations [21,22]. Many drug hypersensitivity reactions were, in spite of intensive search, not HLA-allele restricted. Some previous investigations with SMX and lidocain specific CD4+ T cell clones (TCC), generated from patients with respective allergies, showed that a complete T cell stimulation, as measured through IL-2 secretion, proliferation, and cytotoxicity, required some interaction of the TCR with a HLA molecule, but that the allele could be exchanged [21–23]. Moreover, also the presented peptide could be exchanged [24] without affecting reactivity to SMX. Thus, while drug induced T cell stimulation was clearly HLA restricted, the involvement of a particular HLA-allele was not always documented, as different HLA-alleles (and peptides) could serve as scaffold for full T-cell stimulation [22–24].

**Figure 1:**
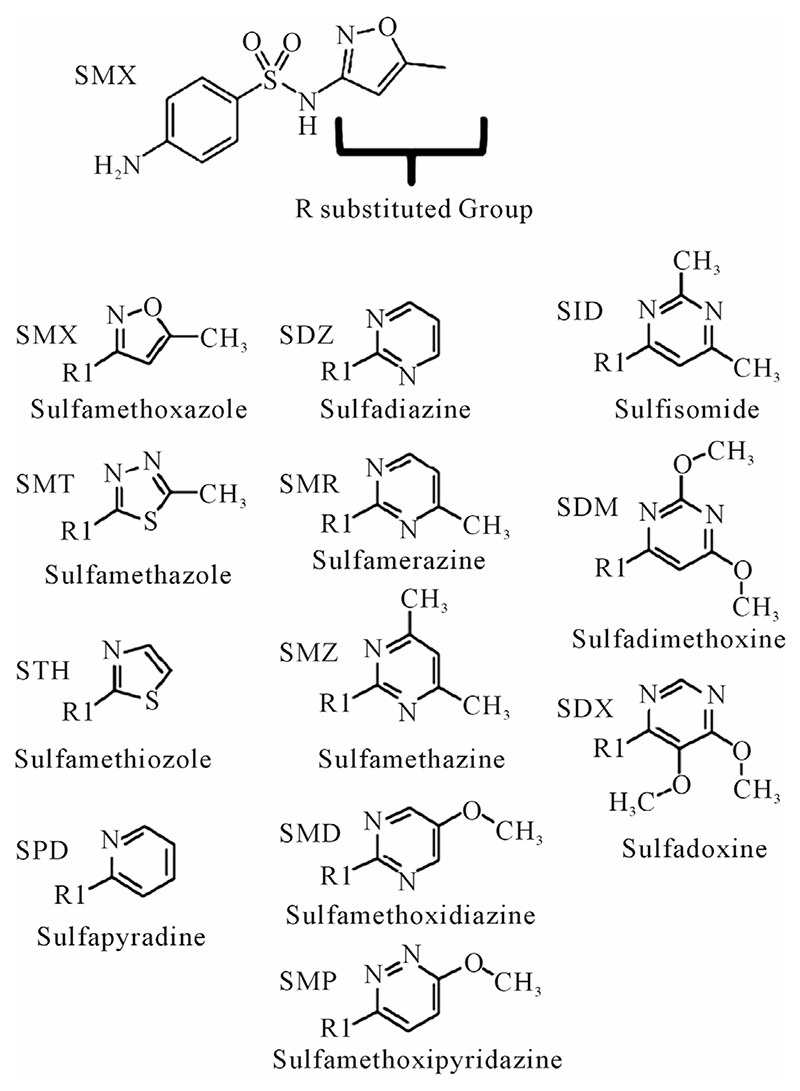
Complete list of compounds (and abbreviations) used in experiments. R substituted group, inserted group shown in table. All R groups are connected to the sulfanilamide core, represented at the top by sulfamethoxazole (SMX), by a single bond to the N of the base sulfanilamide.

Based on these aspects, we pursued the idea that drugs like SMX may interact directly with the TCR, and that this interaction by a non-covalently bound drug can be an essential component of T-cell activation, as it may occur in drug hypersensitivity. To support this concept, we evaluated the stimulatory capacity of SMX and of 11 structurally related sulfanilamides (SA) (Figure 1) with two SMX specific TCC, 1.3 and H13. We investigated the inhibitory capacity of the SMX related compounds for activation of TCCs by SMX itself, using proliferation and Ca++ influx as read out parameters. These SMX reactive TCR of the two TCC were sequenced and 3 dimensional models were generated. Subsequently, we used models to check possible binding sites for SMX and other SA on TCR using docking software. By combining the *in vitro* and *in silico* generated data we could explain on the molecular level the stimulatory or inhibitory activity of SA on these two TCR.

## 2 Results

### 2.1 Differences in Proliferation of SMX Specific CD4+ TCC by Stimulation with 12 Structurally Related Sulfanilamides

**Figure 2:**
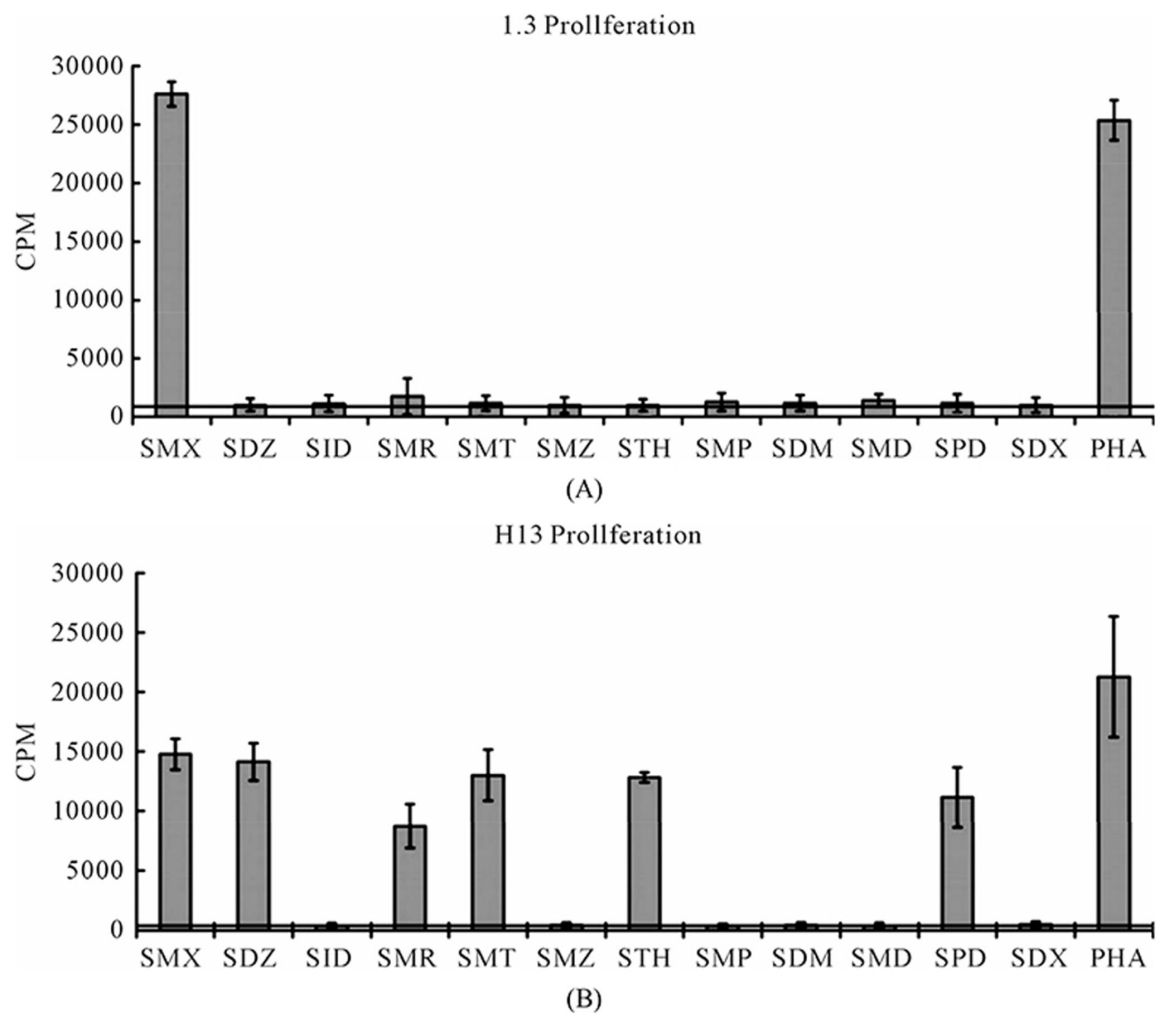
Proliferation assays for two clones. (A) Clone 1.3 and (B) Clone H13. Each represents the mean for 3 - 6 triplicates form different experiments. PHA (phytohaemagglutinin) served as positive control. Standard deviation is shown for each as bars. Solid line crossing Y-axis is mean of background across all experiments represented.

The TCC generated had the CD4 phenotype, were MHC-DRB1*1001 restricted and reacted to concentrations of SMX between 32.5 to 250 μg/ml [4]. T cell receptors of 7 SMX reactive TCC were sequenced (Figure S1). Two TCC, clones 1.3 and H13, were selected for further in vitro analysis, as they could be well expanded in vitro. TCC 1.3 proliferated exclusively with SMX, and was refractory to any stimulation of the 11 additional SA (Figure 1), although they all share the sulfanilamide core structure. In contrast to TCC 1.3, the TCC H13 was able to react to SMX, as well as to similar concentrations of 5 related SA compounds (Figure 2). Six additional SA, however, did not stimulate H13 in the proliferation assays. In addition to distinct cross-reactivity to different SA, the two TCC also differed in the strength of proliferative response, with a weaker response using TCC H13 vs TCC 1.3 (Figures 2(A) and (B)).

### 2.2 Characterizing Drug Induced Ca2+ Influx of TCC 1.3 and H13

According to the p-i model, the drug interacting with the TCR, HLA-molecule or both occurs immediately and independently of prior protein processing or drug metabolism. To confirm that the TCC react to SMX according to the p-i model, we analyzed the very rapidly appearing elevation of intracellular Ca++ of the two TCC upon stimulation by SMX and the indicated panel of tested SA [25]. Both TCC showed a very rapid response with the addition of SMX. H13 reacted only if autologous EBV-B-LCL were used as APC, while use of allogeneic APC not expressing HLA-DR-B1*1001 failed to elicit measurable responses. In contrast, SMX induced stimulation of TCC 1.3 was observed with non-autologous PBMC as well as autologous EBV lines, and also without adding APC, as the TCC were self-presenting [21].

**Figure 3:**
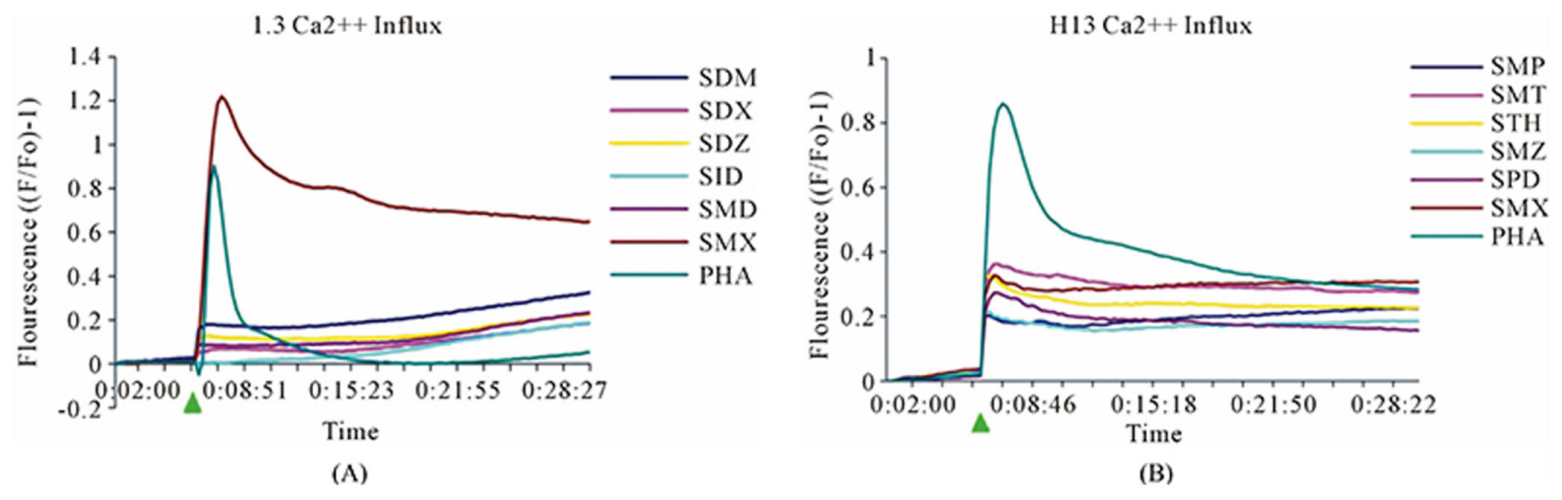
General calcium influx for specified compounds in the TCC 1.3 and H13 (A) Clone 1.3 shows a very strong response only to SMX, equivalent across different experiments and stronger than PHA stimulation. (B) Clone H13. All stimulatory compounds give a response which was weaker compared to PHA. The unresponsive flat lines correspond to the non stimulatory compounds SMZ and SMP. Green arrow, indicates time of injection of each compound.

In clone 1.3 the reaction was specific for SMX only. This SMX induced Ca++ influx was always very strong, similar or even higher than PHA induced influx, and started as early as 14 sec after addition of SMX to the TCC/APC cell suspension. This confirmed a p-i based mechanism (Figure 3(A), Figure S2(A)). Of the other SA, only the SA SMP gave a very moderate response, about one seventh of the strength of SMX, which never resulted in a measurable proliferation (Figure 2(A)). All other compounds showed no Ca2++ influx, which correlated to the proliferation data.

Clone H13 gave a response to SMX and the 5 SA compounds SMT, STH, SDZ, SMR and SPD (Figure 3(B), Figure S2(B), (C)). The same SA were stimulatory in the Ca++ and proliferation assays. The Ca2++ influxes were rapid, occurring within the first minute, again suggesting a direct p-i mechanism. Overall the H13 proliferation to SA was weaker in comparison to those produced by SMX in clone 1.3. The compound SMD gave an inconsistent, weak response in Ca++ measurements, without proliferation, similar to the weak and inconsistent response of 1.3 to SMP.

### 2.3 Inhibition of SMX-Induced T Cell Activation by SA (TCC1.3)

**Figure 4:**
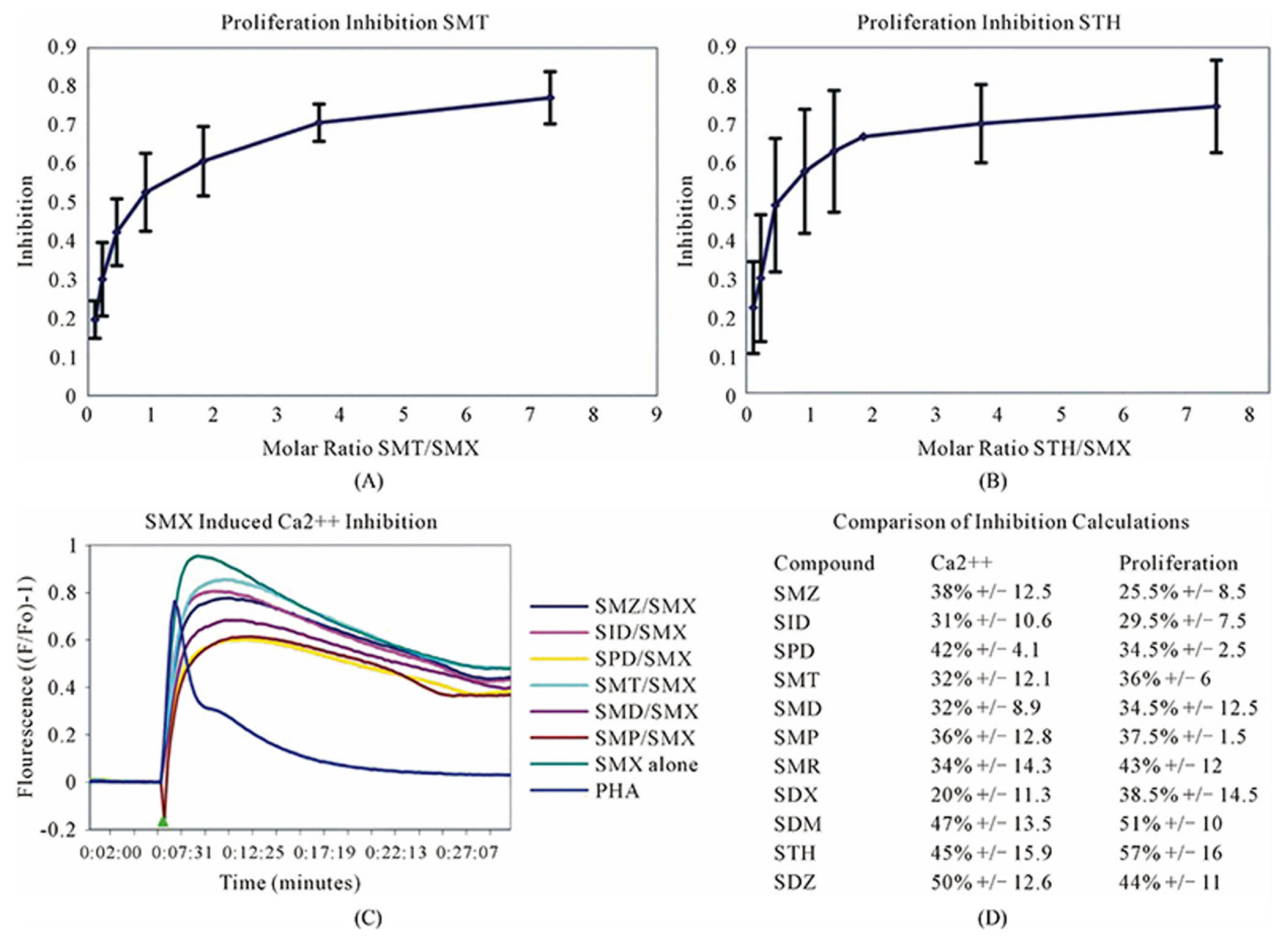
Inhibition of SMX binding by other SA (TCC 1.3). (A) and (B), best and worst inhibition assay profile based on standard deviations (SD) generated respectively. Each represents 3 - 6 independent triplicates in standard proliferation assays across a molar range from 8:1 to 1:8 SMX vs. competitor compound. In 6 compounds tested the curves fit to a single site competition indicating the SMX and the compounds were competing for the same site. (C) Ca2++ influx inhibition for 6 SA indicated. All are at a 1:1 molar ratio of 125μg/ml each. One representative assay is shown from 3 independent experiments with the same results. Green arrow indicates injection time of compounds. (D) Comparison of the calculated inhibition from proliferation and Ca2++ influx at 1:1 molar ratios, specific SA (column 1). Data are shown as % inhibition, with +/− SD, for different assay.

Proliferation assays with SMX and one other SA as competitor were conducted using a range of competitor concentrations. Toxicity assays were preformed first for all compounds, showing that most compounds started to become toxic above 350 ug/ml, with SMX and SMP having a toxicity level above 500 ug/ml before any drug induced cell death was observed (data not shown). Maximal stimulation of TCC 1.3 was reached with SMX at 250 ug/ml, and this was chosen as a maximum total drug concentration for proliferation based competition assays. A minimal measurable response from SMX was determined to be 3 ug/ml giving a rough molar range for testing all competitors from 8:1 to 1:8 competitor to SMX. All mixtures were then tested in triplicate as 7 combined concentrations along this range. Data was then used to generate an inhibition curve, reflected as a percentage decrease in SMX induced proliferation (Figure 4(A), (B) Figure S3). All compounds were found to inhibit SMX induced proliferation of TCC 1.3 in a dose dependent manner. The inhibition ranged from 65% - 80% at a 1:4 molar ratio of SMX to competitor. It did not increase with higher molar ratios significantly. In all cases the generated curves were indicative of a first order competition, showing only a single plateau, however in 5 compounds the standard deviation may cause curve fitting to miss small deviations.

To measure an immediate response from competition of SA an adapted Ca2++ influx was conducted at a 1 to 1 molar ratio of SMX to SA derivative. For this assay, the competitor was allowed to incubate for 5 minutes with the T cell 1.3 and APC, prior to addition of SMX. Without pre-incubation, no consistent inhibition was observed. Our adapted protocol allowed the inhibition calculated for each compound to remain the same, and no difference was observed for calculated inhibition with increased incubation times (data not shown). Influx assays were then generated for each SA against SMX, giving decreased Ca2++ influx with all compounds tested (Figure 4(C), Figure S4). Comparison of calculated inhibition for each compound matched well with those generated by proliferation assays alone in 6 SA derivatives, but varied slightly for the remaining compounds (Figure 4(D)). The immediate inhibition of Ca2++ influx by all compounds according to the protocol used indicates the reaction kinetics related to the SA binding to the TCR are under 5 minutes.

No inhibition was observed for H13: addition of non-stimulatory compounds did not affect the proliferation, addition of stimulatory compounds to low SMX concentrations dose dependently enhanced the proliferation. Data not shown.

### 2.4 Generation of 3D TCR Models and SA Docking

**Figure 5:**
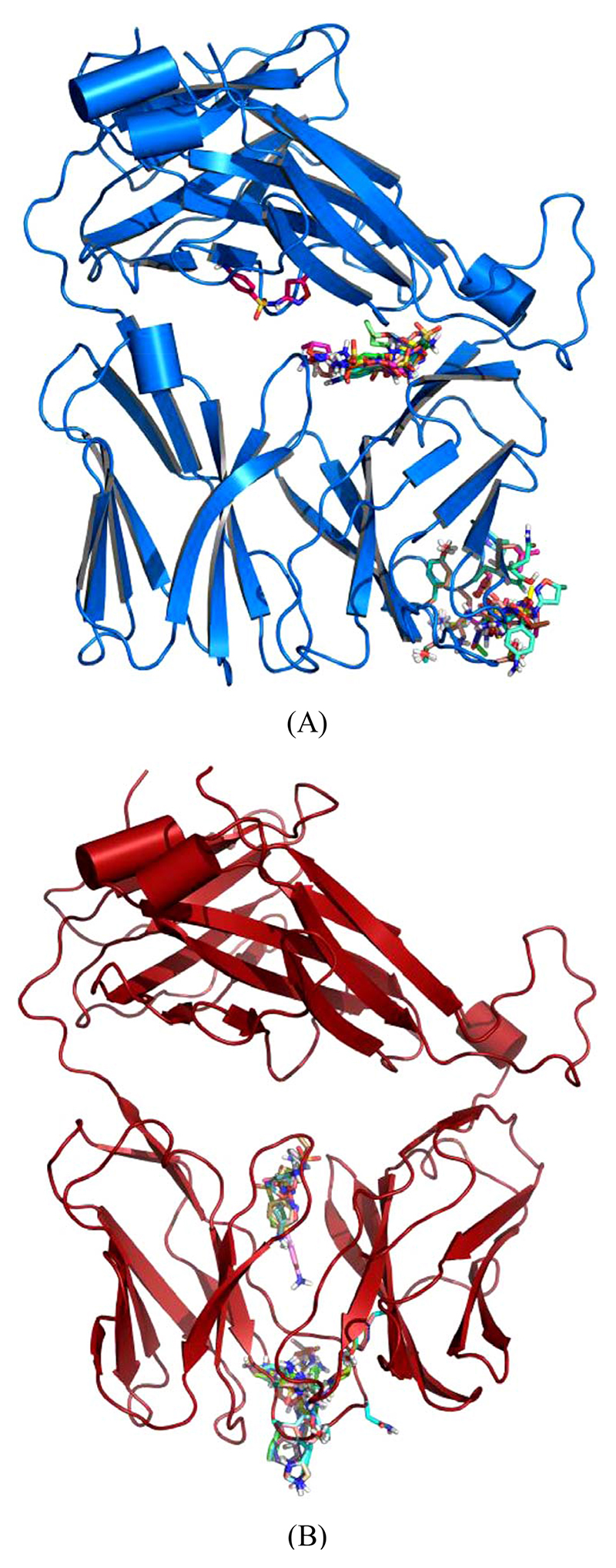
Global Docking of sulfanilamides. (A) TCR from clone H13 (B) TCR from clone 1.3. Shown are clustered SMX at a common site between the Vα and Vβ domains of the TCRs. The secondary clusters at the CDR2β and CDR3α are shown in A and B respectively. A random SMX docking conformation is also shown in A, below the invariant domain, to illustrate random affinities for SMX around the TCR, due to the highly reactive sulfate in SMX.

Models were generated for each of the TCR indicated (Figure S1), as described in methods. In addition, 4 models for non-SMX specific human TCR and a mouse TCR were also generated as controls. Small molecule 3D structures were generated for SMX and each of the SA using chemsketch software and openbabbel to generate 3D coordinates in PDB format. Each small molecule was then docked several times against the individual TCR models globally. Various common binding sites were found across all TCR, including the mouse model (Figure 5). These sites may not be functionally relevant, as they occurred in all TCR models including non-SMX specific TCR and the mouse TCR. The access to some of these sites appears to be blocked by other proteins, such as CD3 subunits and glycosylation [26,27]. Of interest were the additional binding sites on the CDR3α and CDR2β loops, which were found exclusively on the SMX reactive TCR H13 and TCR 1.3.

### 2.5 Docking of SA to the TCR of Clone H13

**Figure 6:**
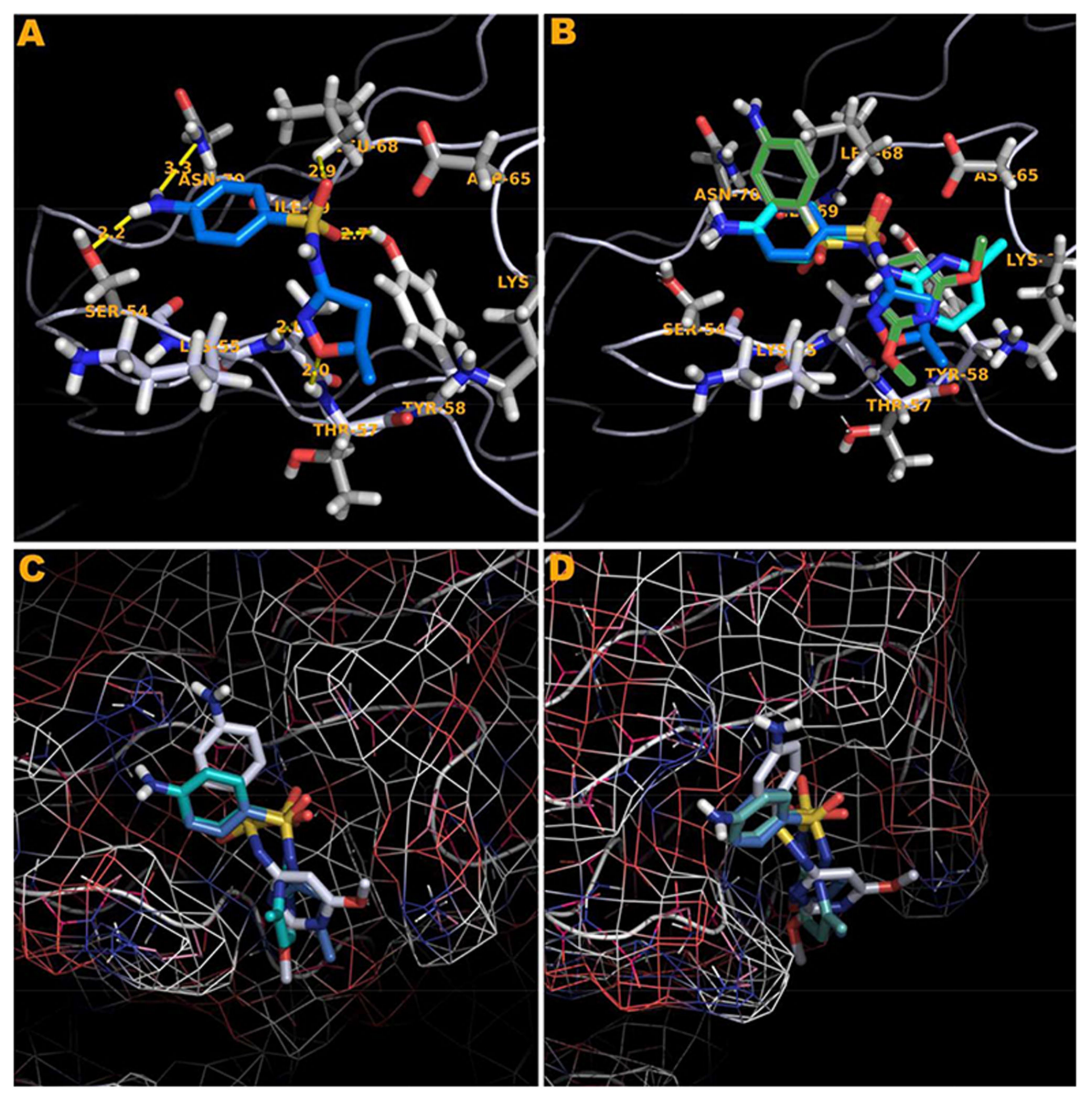
Docking of SA to the CDR2β loop of clone H13. (A) SMX bound in a pocket formed by the residues indicated. 6 hydrogen bonds are shown as yellow dashes, with distances in Å. An additional salt bridge with the SMX sulfur and ASP 65 (top right) occurs at 3.8 Å. (B) The stimulatory SA SMR (aqua) and the non stimulatory SA SDM (green) overlaid with SMX blue. The SA core of SMR and SMX align with less than 0.01 Å difference, while SDM cannot place the domain properly due to steric hindrance from the R group. (C) Surface map with atomic radii colored as nitrogen blue, oxygen red and all other atoms white. SDM white, SMR aqua and SMX blue in the same orientation as in (B) to illustrate the shape of the loop and resulting pocket. (D) same as (C) rotated 90° clockwise to show the depth of the pocket.

The TCC H13 and H25 proliferated to SMX and showed docking of SMX to the CDR2β loop of TCR H13 and H25. Clone H13 was chosen for a more detailed analysis at the molecular level. Using a refined approach, where parameters were set to encompass the CDR2β loop alone, it was found all compounds tested could be made to dock around the suspected site of activity (Figure 6(A), (B)). The SA core of the reactive compounds SMX, SMT, STH, SDZ, SMR, SPD formed interactions with SER 54, TYR 58, LEU 68 and ASN70 of the CDR2β loop. In addition, residues LYS 64 and ASP 65 seemed necessary to make up the overall pocket visible on the CDR2β, along with ASP 65 forming a salt bridge with the sulfur of the SA core itself at 3.8 Å. Two other hydrogen bonds were formed with atoms in the backbone, ALA 56 and THR 57 respectively, which could also be accommodated with amino acid substitutions not causing steric hindrance with the SA R-groups, as the interactions were with backbone atoms alone. In addition, hydrophobic interactions also played a role, by providing an inward force for the R-groups from the amino acids ALA 56, THR 57 methyl group, LEU 68 and to a limited amount ILE 69. Together, these later 4 amino acids make up a hydrophobic region in one half of the CDR2β pocket itself. The strongest interaction was with an oxygen of the SO2 group of the SA core and the terminal OH of TYR 58 which seemed indispensable for the interaction.

In non-stimulatory compounds (SID, SMZ, SMP, SDM, SMD, SDX), the R substituted group size alone prevented the SO2 and NH2 groups from being properly positioned in the CDR2β binding pocket. This was by preventing the proper distance between the TYR 58 SA SO2, where no hydrogen bonding could occur (Figure 6(C), (D)). From superposition of conformations, it was observed that this TYR alone oriented the reactive SA into a tightly bound position. The respective R groups had a small but apparent degree of flexibility in the opposite side of the whole binding pocket. Using flexible docking the non-reactive compounds could dock their R groups into the later site in differing conformations, moving LEU 68 aside by 1 Å. However none could properly place the terminal NH2 or SO2 core of the SA in the same orientation as the reactive compounds simultaneously. This was due to steric hindrance from the larger R groups, moving the SO2 and NH2 away from the loop by only 2 - 3 Å when bound at one end. A fixed angle with the N to S-C bond in the SA core, which has a range 110.0° +/− 2.5 in either direction, was the primary cause [28,29].

Overall in clone H13, SMX was able to form 6 hydrogen bonds and 1 salt bridge bound within the pocket. After energy minimization, these bonds were, SMX to residue respectively, O of SO2 to TYR 58 terminal H 2.7 Å, O of SO2 to LEU 68 Methyl H 2.8 Å, H of terminal NH2 to ASN 70 terminal N 3.2 Å, H of terminal NH2 to SER 54 terminal O 3.1 Å, N ring to ALA 56 NH back-bone 1.9 Å, O ring to THR 57 NH backbone 2.9 Å. A single salt bridge is accommodated by ASP 65 terminal O and the sulfur of the SMX at 3.8 Å. Additional weak interactions with the SA core benzyl ring are re-enforced by LEU 68, and a few weak interactions between LYS 64 terminal NH3 and the pentameric ring carbons of the SMX R group. These ring carbons have a weak negative charge of 0.06 eV due to the cyclic effect of the specific R group, far lower than a hydrogen bond strength but measurable. The other docked compounds which gave a response in the Ca2++ and proliferation assays maintain all the SA core hydrogen bonding, with differences respective to the R substituted groups.

For H13, dehydrated SA showed a much wider degree of docking affinities. By removing the indicated hydrogen alone, it was possible to find an orientation in which all SO2 groups could form a bond with the TYR 58. The overall orientations placed the SA core within or 1 - 2 Å displaced from the SER 54 and ASN 70 site. Main differences were a change in the S-N-C angle from 110° to 127° +/− 2, and the nitrogen charge from almost neutral to −0.4, simply from removing the hydrogen (Supplemental Figure 5(B)).

### 2.6 Docking of SA to the TCR 1.3

**Figure 7:**
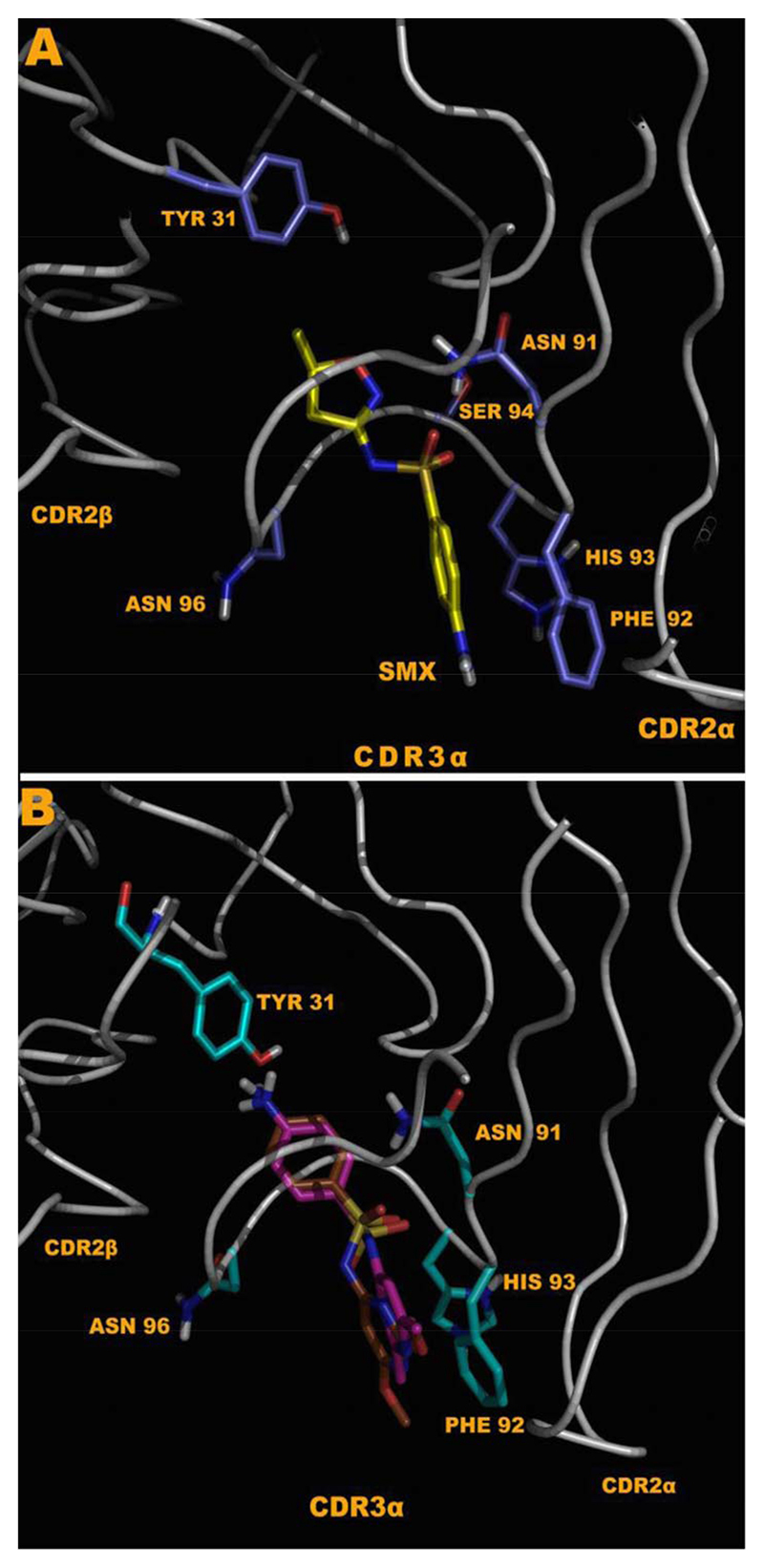
Docking to energy minimized TCR model generated from the sequenced clone 1.3 TCR. (**A**) Shows SMX bound in the CDR3α loop in a reversed conformation, where the SA NH2 is oriented towards the peptide of the MHC peptide groove. (**B**) SID, brown and SMD fuchsia, all SA compounds except SMX and SMP, can bind only in the second conformation. A difference in TYR 31 is shown, as the TYR 31 anchors the compounds via direct hydrogen bonding with the SA core NH2, only via this conformation. In SMX, and SMP there is direct interaction from the R group with backbone N or O, as well as a single hydrogen bond to GLY 95 H.

Using affinity of 6.5 kCal/mol cutoff for positive versus negative docked conformations and as second criterion the involvement of at least 5 hydrogen bonds for each compound, the stimulatory SMX and all other 11 non-stimulatory sulfanilamides were found to dock into the CDR3α loopof clone 1.3. This is compatible with the ability of all 11 SA to block SMX stimulation in functional assays and suggests that competition of SA with SMX for the same binding site accounts for the blocking effect observed in Ca2++ responses, or proliferative capacity. All SA showed a range of between 5-7 hydrogen bonds per compound and direct hydrophobic interactions with PHE 92 of the CDR3α loop. A pi orbital interaction occurred with SMP and SMX in orientations where the SA core unit points to the peptide groove (Figure 7(A)). For all compounds which bound with the SA core oriented away from the peptide groove of the MHC, LEU 99 also provided a hydrophobic interaction for the cyclic portion of the SA core. An energy minimized overlay of all compounds was then conducted to compare differences in binding (Figure S6). All 10 other SA were not able to bind in this reversed orientation, as the size for R groups of the SA derivatives were too large to fit in, or due to charge as described below. This second conformation adopted by all compounds is illustrated (Figure 7(B)), showing the R groups oriented towards the MHC-peptide groove. Overall, two oxygens of the SA SO2 were able to form hydrogen bonds with loop backbone NH. Additional amino specific interactions with the NH of the SA S-N-R neck (Figure 1), along with the O of the SO2 were observed with GLY 95 H and ASN 91 NH2 respectively. In non-reactive SA the NH2 groups of the SA core were always positioned inwards forming a hydrogen bond with the CDR1β TYR 31 (Figure 6(B)). Further docking was conducted in a refined set of dimensions only including the CDR3α loop, yielding the same results.

There is also an effect observable between the hydrophobic groups of the CDR3 loop, which hinder the reversed orientation for a limited set of compounds. This is apparent by looking at SMP and SMD which differ in only one additional charge (Figure 1). SMP has a complete benzene like hydrophobic side to the R group, while SMD has a nitrogen with a large negative charge of 0.4 to 0.5 due to the cyclic nature of the ring. This nitrogen would interact with the loop rather than passing through until the SO2 core is recognized. SMP is able to use hydrophobic interactions with THR 97 and LEU 99 to push the R group through into an opposite orientation.

Docking of all compounds in a dehydrated form (Supplemental Figure 5(A)) only lowered overall docking affinities, mostly by disruption of the PHE 92 hydrophobic interaction, and slight movement of the SO2. In the non-dehydrated SA the SO2 was always oriented in the CDR3α loop center with a repulsive effect from the hydrogen of the SA neck from loop backbone oxygens and positive NH backbone atoms (Figure S5(A)). Dehydrating the SA adds an additional negative charge in this interaction. Loop residue ASN 91 made up a net difference for loss of 1 O to NH interaction by moving hydrogens towards one O of the SO2. This shows computationally the SA SO2 core is the primary recognized portion of the molecule by the CDR3α loop.

### 2.7 Further T Cell Clone and TCR Characteristics

The two TCC analyzed were CD4+, expressed the skin homing receptor CCR4 but not CCR10, were perforin positive and expressed CD107a upon activation by SMX (Figure S7). For clone 1.3 the CD107a upregulation is maximal at 2 hours post stimulation with SMX. Inhibition of this up-regulation of surface expressed CD107a was observed using STH for clone 1.3 (Figure S7(C)). At approximately a 1 to 1 molar ratio STH was able to diminish degranulation in clone 1.3 by 50% at 2 hours. Overall, the competition may have only delayed the up-regulation, as time points at 4 hours indicated a slight increase in CD107a with STH/SMX induction indicating either a prolonged competitive binding and lower induction, or only lower initial stimulation.

## 3 Discussion

In this study we pursued the concept of direct drug interactions with immune receptors. We used the model of SMX hypersensitivity and respective TCC to analyze a stimulatory potential of direct SA binding to certain TCR. We took advantage on previous studies of SMX specific TCC, which had already revealed their dependence on APC for optimal stimulations and that different TCC, with structurally different TCR, show quite distinct cross-reactivities with up to 11 additional SA [4,30]. In this study we focused on two distinct TCR/TCC (1.3 and H13), which showed distinct cross-reactivities to 11 SA and correlated their functional, structural, and inhibitory in vitro data with computational docking data. This combined analysis allowed to locate the SMX binding site to either the CDR3α for 1.3 and to CDR2β for H13 and to explain the different functional and inhibitory capacities of the SA on the two TCR/TCC 1.3 and H13^1^.

The TCC used are characterized as “p-i-clones”, meaning that their stimulation was not dependent on prior drug metabolism and presentation of a SMX-modified peptide by APC. Such p-i interactions of drugs are often very labile, and washing the cells,T-cells, APC or both, already abrogates reactivity [5]. The only exception with rather stable binding is Abacavir [13,17], as this compound has been shown to bind with sufficient affinity to the HLA-B*57:01 molecule without being removed by washing. This lability of drug binding to immune receptors make it difficult to analyze the p-i concept by biochemical means or use e.g. of radioactive labeled drugs, as these methods all require washing steps.

Both TCC showed a rapid Ca++ influx after adding the compounds to TCC in the presence of APC. This immediate reactivity for both clones was even faster thanthe TCC reactivity of high affinity abacavir reactive TCC, which reacted after ca. 60 - 100 sec [13]. This rapid Ca++ influx is similar to other drug-receptor interactions, where signaling occurs within seconds rather than hours for non-channel based receptors. We opted to perform the analysis with TCC instead of transfected cell lines, as the reactivity of TCC was substantially higher. All assays were done with non-toxic concentrations, mostly with 250 μg/ml of SMX or related SA.

While both TCC reacted rapidly to SMX with immediate Ca++ influx, the two TCC H13 and 1.3, are quite different if analyzed in detail. As reported previously, using TCR transfected cell lines, which are devoid of self HLA-molecules, *full* activation by SMX, reflected as IL-2 secretion or proliferation, required not only the presence of the drug, but also an interaction with HLA-molecules on APC [23,31,32]. In the case of TCC H13 the HLA-DR-B1*1001 molecule, provided by autologous EBV transformed B cells alone, was a requisite. In contrast, the TCC 1.3 reacted to SMX regardless of PBMC type used, as it was self-presenting.

A clue to an in depth analysis of the two TCC was their different reactivity to 11 other SA. Their responses in the T-cell assays were only due to cross-reactivity of the originally SMX specific TCC, and not due to prior exposure to the SA, which are mostly not available as drugs or only for veterinary use. TCC 1.3 proliferated only to SMX, while the other 11 SA were not stimulatory, even if their molecular structure was rather similar (Figure 1). Ca++ influx measurements confirmed the exclusive SMX reactivity, with the exception of the SA SMP, which stimulated a minor Ca++ influx, but no proliferation. Interestingly, all non-stimulatory SA did inhibit the proliferation or the Ca++ influx to SMX stimulation in TCC 1.3. In the proliferation assays, the inhibition was maximal with ~50% - 70% at a 4:1 ratio of SA/SMX. The inhibitory effect was dose dependent and achieved at non-toxic concentrations of SMX and competing compounds together. Ca++ influx was also inhibited, but only if the blocking SA was added 5min before SMX. The inhibition was averaging 27% - 37%, and consistent with that calculated by proliferation inhibition curves at the same SA/SMX ratio.

The TCC H13 reacted with SMX and 5 additional SA, while 6 other SA failed to stimulate this TCC. The non-stimulatory compounds did not inhibit SMX reactivity in competition assays, because their intrinsic stimulatory activity was very similar to the stimulatory activity of SMX. Thus, the stimulatory compounds increased the effect of SMX in a dose dependent way (data not shown).

As drug and HLA interactions seem to be involved in full T cell stimulation, it is possible that SMX and the other SA bind first to the HLA, and are then recognized by the TCR. However, our data argue strongly against first HLA-binding (actually HLA-DR-B1*1001). All SA and SMX have shown some functional involvement, either stimulatory or blocking, and these differences cannot be explained by DR-B1*10:01 binding, which is not different between TCC 1.3and H13. E.g., the SA STH stimulates H13, but does not stimulate 1.3:If this would be explained by binding to HLA-DR-B1*1001, one has to postulate that STH has no binding sites on the TCR 1.3, while the TCR H13 binds STH. But STH was able to block SMX (1.3), and could stimulate (H13). Or the SA STH andSMTare both able to block the SMX induced stimulation of TCC 1.3. However the reaction of H13 to SMX is not blocked, and STH and SMT are either stimulatory (STH) or gives no response (SMT), a constellation which is not compatible with the hypothesis that both SA would compete with SMX binding on HLA-DR-B1*1001. Therefore, the reactivity of competing SA differs depending on the TCR, not on the HLA site, which is identical for both TCC.

[31,32] To explain the differences in the reactivity of H13 and 1.3 to SMX and related SA as well as to understand the differences in blocking SMX induced stimulation by SA we applied docking models for the SA and TCR. Initial SMX docking experiments showed that SMX and other SA might bind to a number of sites on the whole TCR. All of these global binding sites are outside the antigen-specific interaction site facing the MHC-peptide complex, and whether they are accessible to drugs is unclear. It is likely that these possible dockings occur via the sulfur moiety in the SA, which can form non-covalent interactions with a multitude of amino acids on protein surfaces without a functional consequence, as non-specific binding of this type has been shown to occur with similar compounds.

Docking revealed two unique sites on either TCC H13 or 1.3, characterized by 5 - 7 hydrogen bonds, which were absent on the TCR without SMX specificity. They were identified on the CDR2β of TCR H13 and on CDR3α of TCR 1.3. How can one be sure that these sites are relevant? As we used TCC instead of TCR transfected cell lines, which had a better discriminatory power than TCR transfected cell lines, we took the approach to use distinct SA to define the possible binding site. This proved to give rather clear results, and helped discriminate HLA or TCR interactions. The CDR2β binding site for SMX on the H13 TCR was capable of binding the 5 additional stimulatory SA, while the 6 non-stimulatory SA did not bind. Thus, the docking data were in agreement with the functional data and could thus explain the selective stimulation by SMX and the 5 SA.

With TCR 1.3, docking revealed 7 hydrogen bonds to SMX on the CDR3α region, with almost an antibody like recognition of the compound [33]. The other 11 non-stimulatory SA were also binding to this site, with an equal affinity. This overlap in binding sites could explain the blocking effect of the 11 SA on SMX induced proliferation and on Ca++ influx. However, it did not explain why SMX was stimulatory, while the other SA were not. A closer look at SMX and SA binding in TCC 1.3 revealed subtle, but possible relevant differences. Overall only two compounds were eliciting a measurable Ca2++ response, while the rest of SA did not. Both Ca2++ influx inducing SA, SMX and SMP, were the only SA able to bind in two orientations to the CDR3α loop. All the non-stimulatory SA contained an R groups, which prevented an orientation, where the terminal NH2 of the SA core was oriented towards the peptide groove. Thus SMX and SMP might be stimulatory in Ca2++ influx, because they were the only compounds able to interact with the TCR differently than the other SA. Why only SMX induces proliferation needs to be evaluated further.

For clone H13 two separate characteristics of the compounds and their ability to bind in specific orientations seem to be important, which may also shed light on more detailed functioning of the TCR at the amino acid level. In the 6 non-stimulatory SA (SID, SMZ, SMP, SDM, SMD, and SDX), the size of the R group hinders the SA core to positioning itself in the orientation depicted in Figure 6(A). The overall distance of the SO2 of these non-stimulatory SA is moved from the pocket by only 1 - 2 Å. This small shift moves an O of the SO2 away from a proper orientation against the TYR 58, which seems to be indispensable in anchoring the SA core within the loop. Interaction with TYR 58 has been postulated as important on MHC class 1 TCR interactions via mutational studies, as well as invariant chain TCR T cells [32–35]. Another aspect was that the terminal NH2 of the SA cannot be properly placed, if the angle of the S-N-R in the SA is not correctly bent. Our computational results show that by modeling similar structures and their angles in this neck region, changing the bend from 110° to 127° allows all compounds to dock into this site. Respective R groups bind in the end portion above THR 57 (Figure 6(C), (D)), for all SA but fail to be able to simultaneously place the SA core in the other end of the pocket. Thus, size of the R group and angle of the S-N-R influence the ability to bind to H13.

Our data suggest that drugs like SMX and other SA may interact directly with TCRs and that they can induce T cell activation if an additional interaction with the HLA molecule is provided [23,30]. Actually, the SMX binding may alter the interaction of the TCR with pMHC, as shown by computational analysis of TCR H13§. The drug binding has to occur at certain, functionally relevant sites of the TCR, such as the CDR3 or CDR2 sites. Structurally related molecules may also bind: they may signal as well, or may just bind, without signaling, or may block signaling of a competing SA. This means that computational studies may reveal a possible binding site, but must be followed by functional analysis, as drug binding does not necessarily lead to signal transmission.

The consequences of these findings are multifold. With regard to *drug hypersensitivity*, the starting point of our investigations, our data underline the p-i concept and their two subforms. In one model, p-i (HLA), drugs preferentially interact with certain HLA molecules [13, 17–19]. These reactions are exemplified by strong HLA-allele restricted hypersensitivity reactions. Immune stimulation in this model occurs by the reactive T cell, which itself is not altered by the drug, but possesses a TCR able to interact with the MHC-drug-peptide complex. As shown in the carbamazepine model, only if such T-cells are present in the circulation, the carbamazepine-MHC complex is harmful [36] In the abacavir model, all individuals possess reactive TCC/TCR, which recognize the abacavir-MHC-peptide complex [12] or altered peptides [17–19]. It is a more indirect stimulation of T cells.

In the p-i (TCR) model, an initial interaction of the drug with a peculiar TCR occurs.It is a more direct effect of the drug on the reactive cell, the T cell. The details of the drug induced TCR signaling are not yet clear: The MHC/peptide may act as scaffold and/or the SMX binding to TCR may result in some change of the TCR which results in better binding to pMHC§.

The *immunological consequences* of our data are also multifold. The data illustrate that the drug binding regions of the TCR show an extreme fine specificity and fine tuning for signaling. Our findings may provide further tools to decipher this still enigmatic area of TCR signaling and specificity. These studies also show that TCR-MHC interactions might be amenable to usual pharmacological tools like blocking of a receptor by a competing ligand.

The p-i TCR concept implies that the T cell stimulated by a drug in adverse reactions also have a peptide specificity, which is mostly unknown. Preliminary data with allo- and simultaneously drug reactive T cells have indeed revealed that an allo-reactivity can be blocked by a drug (unpublished). As the data always refer to only one single TCC/TCR, and another TCC behaves differently, a therapeutic intervention (e.g. blocking a certain TCR) could be imagined only on clonal expansions, since other TCC would react differently to the same drug. An example is the SA STH: it is inhibitory for SMX stimulation by TCC 1.3, but the same SA further enhances the SMX triggered stimulation of H13, which comes from the same individual.

In conclusion, drug hypersensitivity reactions may not only develop because drugs are binding to HLA but also on drug binding to TCR. These are highly interesting models for “unusual” T cell stimulations and may open new possibilities of pharmacological interventions with the specific immune system. In addition, drug interactions may prove a means to further understand functionality of immune receptors.

## 4 Materials and Methods

### 4.1 T Cell Clones

The PBMC, EBV B cell lines and T cells were derived from a patient with exanthema, malaise and well documented SMX specific T cell proliferation in vitro [4,37]. Cloning was performed by limiting dilution as described previously. To test specificity of the clones, T-cells were analyzed by means of proliferation, with 250 μg/ml SMX. The specific TCC were expanded further by PHA-stimulation (1 μg/mL; LaboratoiresEurobio, Cortaboeuf-Cedex B, France) in combination with allogeneic, irradiated PBMCs every 14 days. IL-2 was added after 24 hours to a concentration of 100 IU final volume, and then every 48 hours for 14 days. Both TCC required interaction with HLA and H13 required autologous APC like EBV-B-LCL and did not react to addition of allogeneic APC. TCC 1.3 was also stimulated weakly without adding APC in prior studies, and robustly with the addition of non-autologous APC, or HLA-DR-B1*1001 transfectants.

### 4.2 DNA Sequence Alignments

DNA sequences obtained from sequencing of the reactive clones were translated using DNA to Protein from EMBL translation tool (http://www.ebi.ac.uk/Tools/st/). These were then used to obtain the IMGT (http://www.imgt.org) nomenclature used by the NIH blastp protein sequence searching (http://blast.ncbi.nlm.nih.gov) using the IMGT database as a template. Further sequence alignments were made with all respective clone sequences for the *α* and *β* domains of each sequenced TCR using clustalW multiple sequence alignment.

### 4.3 Proliferation Assays

Briefly, 25,000 TCC and 30,000 irradiated PBMCs or EBV-B-LCL were incubated together per well with 250 ug/ml of the indicated compounds, medium alone (CM) or PHA (1 ug/ml). 3H-thymidine was added after 48 to max 72 hours for exactly 12 hrs and proliferation measured via scintillation counting (Top Count, Perkin Elmer). Averages of at least three and up to six runs for each experimental condition were collected and all means along with statistical differences were calculated. The SA compounds are listed in Figure 1 and are all obtained from Sigma-Aldrich. None of the chemicals showed a toxic effect if used <350 μg/ml.

To test for inhibition of proliferation, the total of two compounds, SMX and competitor, were added at the same time, with a standard proliferation assay as above. All compounds tested in proliferation assays were also used in the competition assays. Both compounds together were not exceeding 250 μg/ml in total. The range of competitive testing was 8:1 - 1:8 (molar ratios). The averages of at least three independent runs were plotted per molar ratio across seven different ratios within the stated range, for each point plotted.

### 4.4 Calcium Influx Assay

TCC were incubated for 10 minutes, and spun down twice in RPMI w/o Phenol red and then incubated with 2 μg/ml Fluo-4 AM (Invitrogen, Carlsbad, CA) in RPMI w/o Phenol red (Sigma Chemicals Co, Buchs, Switzerland) at 37°C for 30 min. After incubation, cells were washed twice with and resuspended in RPMI w/o Phenol red. Cells were plated in half-area clear bottom 96 well plates (Corning, LifeSciences, CA, USA) at 1 × 10^5^ cells per well (5 × 10^4^ TCC and 5 × 10^4^ irradiated APC or EBV) spun for 1 minute at 1500 RPM’s, and further incubated for 20 min at 37°C in dark to allow cells to equilibrate and uniformly cover the plate surface. Measurement was performed on a Synergy-4 instrument (BioTek Instruments Inc., Highland Park, VT, USA) at 37°C with an excitation band of 485/20 nm and fluorescence was measured at 528/20 nm. Baseline signal (F_0_) was recorded during 5 min before the addition of antigen solutions and subsequent fluorescence measurements were performed for 60 min. PHA stimulation (1 ug/ml final well concentration) or ionomycine at 1 ug/ml served as positive control. The results are shown as normalized fluorescence (F/F_0_)-1. Averages of at least three independent runs for each compound were tested per T cell clone used with similar results.

In competition assays, the final combined concentrations of each competitor and SMX were 250 ug/ml to avoid toxicity, at a 1 to 1 ratio. To reduce or eliminate variation observed without pre-incubation in calculated inhibition, the competing compounds were added with a multi-pipette at the start of the calcium reading, and SMX added 5 minutes later.

### 4.5 Flow Cytometry

All fluorescent conjugated or unlabeled monoclonal antibodies were purchased from BD (Becton, Dickinson and Company. NJ, USA) or Biolegend (San Diego, CA, USA). For all standard staining, cells were plated in V bottom, 96 well plates at 200 K TCC, or 200 K TCC and 200 K PBMC as indicated. Cells were dyed for 30 minutes at 4°C in dark, and washed 3 times all on ice with chilled PBS before reading. All data points are at least 3 readings from independent wells, on 1 - 2 separate days, using a BD FACSCanto™. They were analyzed using FACS DIVA software.

In CD107a expression up-regulation kinetic experiments, cells were plated at 200 K TCC and 200 K PBMC per well in normal RPMI supplemented with heat inactivated 10% FCS. Either the indicated compound, PHA 1 μg/mL or nothing as a negative control were added [38]. Competition of CD107a upregulation by STH was evaluated with the final concentration of SMX/STH at 125 ug/ml each. SMX at 125 ug/ml run in parallel was used as a control. For all, at least 3 independent reads were conducted per time step across 3 different experiments. Means of these were then graphed, with standard deviation represented by error bars.

### 4.6 Model Generation

All protein models were generated using automated model building via Swiss-modeller web site (http://swissmodel.expasy.org/) [39]. In addition to this, models were analyzed using O molecular software [40], and further energy minimized using Gromacs, gromos53a6 force field as necessary to generate the final starting structures [41]. For TCRs, the DNA sequences of each TCR *α* and *β* domain from 8 separate SMX reactive clones were used against the highest resolution TCR X ray structures found in the PDB database, ranging from 1.5 to 2.6 Å based on overall amino acid homology (PDB codes 1KGC, 1FYT, 1MI5). The generated TCR *α* and *β* subunits were then aligned in O against the PDB 1FYT using the MHCII-peptide in the structure as well, and further energy minimized in water solevent at 300 K until all amino acids fitted a favorable, non-sterically inhibited conformation.

As controls for docking, random poly alanine TCR *α* and *β* sequences or TCR from a T-cell non-reactive to SMX, were generated as above. A second control, we used a single TCR *α* and *β* from an *M. musculis* found in Uniprot A2NTU7 and Q7TND8, with models generated as described. Sequences are shown in supplemental Table 1 for all reactive clones used to generate initial models. All were aligned to the closest matching PDB listed above, and energy minimized to eliminate errors from alignment in bond lengths or angles.

Small molecules used in all models, Docking or Molecular Dynamics were generated using simple sketch and conversion software. In short, the compound structure was drawn into ACDlabsChemsketch (Advanced Chemistry Development, Toronto, Ontario, Canada) to generate a MDL mol file. These files were then converted to PDB files with 3D coordinates using OpenBabel (GNU public license) freeware. As these were small molecules, energy minimization prior to using them in Docking runs was conducted using simple Chimera Software on a PC (UCSF Chimera package from the Resource for Biocomputing, Visualization, and Informatics at the University of California, San Francisco).

### 4.7 Docking

All docking was performed using AutoDock or Auto-DockVina, used as cross comparisons [42,43]. The later software is an optimized version of AutoDock for parallel processor execution. In all Docking runs, at least 6 rigid and 6 flexible docking runs were conducted for the final analysis. Each run constitutes 10 individual dockings of each compound used. An initial approach was to conduct Docking runs using a spars matrix approach encompassing the lower 2/3 of the TCR, and then limiting runs to regions of the TCR where clustering was observed. Clustering included 3 or more docked conformations in the same region or area within 3 - 4 Å of each other from at least 3 sparse matrix runs. These limiting runs were then further refined to determined sites showing clear affinities, and at least 6 limited runs were conducted. For flexible docking, amino acids in the CDR3 or CDR2 loops were allowed to move freely, including backbone flexibility as allowed by AutoDock or AutoDock-Vina. For this later, at least 6 additional flexible docking runs were conducted. For each TCR model, the compound library containing all SA was screened against the finalized sites of interest. A cutoff for positive versus negative docked conformations for all molecules was generated for this work alone. The cutoff is based on underestimation of full hydrogen bonding energies, and takes into account only an arbitrary mean based on docking output scores, total hydrogen bonds along with initial visual inspection. The resulting scoring cutoff is 6.5 kCal/mol +/− 0.2, which corresponds to at least 5 hydrogen bonds [42,44].

### 4.8 Model Analysis

All final docked ligand models, or model comparisons were analyzed using multiple software to check for validity. Software used including Pymol ((Schrodinger, LLC (2010) The PyMOL Molecular Graphics System, Version 1.3r1)), Gromacs and AutoDock Tools, all of which have internal analysis tools to measure atomic distances and energies produced from Docking software, and internal hydrogen bonding or salt bridging based on distances and angles [45]. Models were also inspected visually using Pymol [45]. Surfaces based on atomic radius’s were also generated in Pymol based on AMBER internal tables of atomic radius for both docked small molecules and the respective TCR, and inspected visually. All figures of protein models were generated with a freeware Pymol utility rendering script along with the Pymol graphics system.

### 4.9 Statistical Analysis

Statistics were performed using qtiplot (Copyright © 2004-2011 Ion Vasilief and Stephen Besch). Briefly, all proliferation, proliferation inhibition and FACS data were fed into the software using spreadsheets, and standard error, means and standard deviations plotted for individual points in all graphs. One way ANOVA was performed on plotted data, and significant differences scored as <0.05 >0.001, *, or <0.001, ** for respective calculated P values.

## Supplementary Material

Supplementary Material

## Footnotes

1 The SMX binding site on H13 were further analysed using functional dynamics (St. Watkins and WJ Pichler: Sulfamethoxazole Induces a Switch Mechanism in T cell Receptors Containing TCRVβ20-1 by Altering pHLA Recognition, PLOS one, submitted.

## Abbreviations

SMX: sulfamethoxazole
SA: sulfanilamides, p-i concept: pharmacological interaction concept
TCC: T cell clones
TCR: T cell receptor for antigen
MHC: maior histocompatibility complex
pMHC: peptide-MHC
CDR: complementary determining region
