# Supplementary material for: Activating interactions of sulfanilamides with T cell receptors*
Source: Open J Immunol. Author manuscript; Available in PMC 2022 Sep 27. (PMC7613643; doi:10.4236/oji.2013.33019)
Supplement: Supplementary Material [file EMS154408-supplement-Supplementary_Material.pdf]

SUPPLEMENTAL

```
Sequenced TRAVS used
Clone TRAV (IMGT nomenclature)          Sequence

8.5_TRAV 8-4        MLLLLVPVLEVIFTLGGTRAQSVTQLGSHVSVSEGALVLLRCNYSSSVPPYLFWYVQYPN
8.18_TRAV 8-3       MLLELIPLLGIHFVLRTARAQSVTQPDIHITVSEGASLELRCNYSYGATPYLFWYVQSPG
NO2-TRAV 2          MASAPISMLAMLFTLSGLRAQSVAQPEDQVNVAEGNPLTVKCTYSVSGNPYLFWYVQYPN
H25_TRAV  3         MASAPISMLAMLFTLSGLRAQSVAQPEDQVNVAEGNPLTVKCTYSVSGNPYLFWYVQYPN
N3_TRAV 1-1         ---MWGAFLLYVSMKMGGTAGQSLEQPSEVTAVEGAIVQINCTYQTSGFYGLSWYQQHDG
1.3_TRAV 12-2       -----------------------------SVFQEGAIVSLNCTYSDRGSQSFFWYRQYSG
NO3_TRAV 9-2        MNYSPGLVSLILLLLGRTRGDSVTQMEGPVTLSEEAFLTINCTYTATGYPSLFWYVQYPG
H13_TRAV17          --------------------QQGEEDPQALSIQEGENATMNCSYKTS-INNLQWYRQNSG
                                                *     :.*.*        : ** *   .

8.5_TRAV 8-4        QGLQLLLKYTSAATLVKGINDFEAEFKKSETSFHLTKPSAHMSDAAEYFCAVPDGG-PMR
8.18_TRAV 8-3       QGLQLLLKYFSGDTLVQGIKGFEAEFKRSQSSFNLRKPSVHWSDAAEYFCAVGALTTDSW
NO2-TRAV 2          RGLQFLLKYITGDNLVKGSYGFEAEFNKSQTSFHLKKPSALVSDSALYFCAVRDKAG-GY
H25_TRAV 3          RGLQFLLKYITGDNLVKGSYGFEAEFNKSQTSFHLKKPSALVSDSALYFCAVRDKAG-GY
N3_TRAV 1-1         -GAPTFLSYNGLDGLEETGR-FSSFLSRSDSYGYLLLQELQMKDSASYFCAVCSGNT--G
1.3_TRAV 12-2       KSPELIMSIYSN-GDKEDGR-FTAQLNKASQYVSLLIRDSQPSDSATYLCAVNFHSG--N
NO3_TRAV 9-2        EGLQLLLKATKADDKGS-NKGFEATYRKETTSFHLEKGSVQVSDSAVYFCALSNQAG---
H13_TRAV 17         RGLVHLILIRSNEREKHSGR-LRVTLDTSKKSSSLLITASRAADTASYFCATDG-----N
                     .   ::            :            *         *:* *:**

8.5_TRAV 8-4        GKSTFGDGTTLTVKPNI-------------------------------------------
8.18_TRAV 8-3       GKLQFGAGTQVVVTPDI-------------------------------------------
NO2-TRAV 2          QKVTFGIGTKLQVIPSKWI-----------------------------------------
H25_TRAV  3         QKVTFGIGTKLQVIP---------------------------------------------
N3_TRAV 1-1         -KLIFGQGTTLQVKPDI-------------------------------------------
1.3_TRAV 12-2       TPLVFGKGTRLSVIANIQNPDPAVYQLRDSKSSDKSVCLFTDFDSQTNVSQSKDSDVYIT
NO3_TRAV 9-2        TALIFGKGTTLSVSS---------------------------------------------
H13_TRAV 17         -QFYFGTGTSLTVIP---------------------------------------------
                        ** ** : * .
```

(A)

```
Sequenced TRBVS used
Clone TRBV (IMGT nomenclature)          Sequence

H25_TRBV 2          MDTWLVCWAIFSLLKAGLTEPEVTQTPSHQVTQMGQEVILRCV-PISNHLYFYWYRQILG
1.3_TRBV 9          MGFRLLCCVAFCLLGAGPVDSGVTQTPKHLITATGQRVTLRCS-PRSGDLSVYWYQQSLD
8.5_TRBV 27         ---------VVLCLLGAPLEAQVTQNPRYLITVTGKKLTVTCS-QNMNHEYMSWYRQDPG
8.18 TRBV 19        ----------SLSLGATPWMVESLS-PKYLFRKEGQNVTLSCE-QNLNHDAMYWYRQDPG
NO2-TRBV 2          MDTWLVCWAIFSLLKAGLTEPEVTQTPSHQVTQMGQEVILRCV-PISNHLYFYWYRQILG
N3_TRBV 4-3         MGCRLLCCAVLCLLGAVPMETGVTQTPRHLVMGMTNKKSLKCE-QHLGHNAMYWYKQSAK
NO3_TRBV 5-1        MGSRLLCWVLLCLLGAGPVKAGVTQTPRYLIKTRGQQVTLSCS-PISGHRSVSWYQQTPG
H13_TRBV 2-1        -------------------GAVVSQHPSRVICKSGTSVKIECRSLDFQATTMFWYRQFPK
                                              . *   .         : *          . **:*

H25_TRBV 2          QKVEFLVSFYNNEISEKSEIFD-DQFSVERPDGSNFTLKIRSTKLEDSAMYFCASSEAYS
1.3_TRBV 9          QGLQFLIHYYNGEERAKG-NIL-ERFSAQQFPDLHSELNLSSLELGDSALYFCASSVDAD
8.5_TRBV 27         LGLRQIYYSMNVEVTDKG-DVP-EGYKVSRKEKRNFPLILESPSPNQTSLYFCASSLR-R
8.18 TRBV 19        QGLRLIYYSQIVNDFQKG-DIA-EGYSVSREKKESFPLTVSAQK-NPTAFYLCASSIG--
NO2-TRBV 2          QKVEFLVSFYNNEISEKSEIFD-DQFSVERPDGSNFTLKIRSTKLEDSAMYFCASSEAYS
N3_TRBV 4-3         KPLELMFVYSLEERVENN-SVP-SRFSPECPNSSHLFLHLHTLQPEDSALYLCASSRQTG
NO3_TRBV 5-1        QGLQFLFEYFSETQRNKG-NFP-GRFSGRQFSNSRSEMNVSTLELGDSALYLCASSLLQG
H13_TRBV 20-1       QSLMLMATSNEGSKATYEQGVEKDKFLINHASLTLSTLTVTSAHPEDSSFYICSARGQGE
                      :  :      ||| |    .      |           : : :     :: *:*::

H25_TRBV 2          N--QPQHFGDGTRLSIL-------------------------------------------
1.3_TRBV 9          ----TQYFGPGTRLTVLEDL----------------------------------------
8.5_TRBV 27         DF-YEQYFGPGTRLTVTEDL----------------------------------------
8.18 TRBV 19        ---NEQFFGPGTRLTVLEDLKNVFPPEVAVFEPSEAEISHTQKATLVCLATGFYPDHVEL
NO2-TRBV 2          N--QPQHFGDGTRLSIL-------------------------------------------
N3_TRBV 4-3         TGGTKLFFGSGTQLSVLEDL----------------------------------------
NO3_TRBV 5-1        N--TEAFFGQGTRLTVV-------------------------------------------
H13_TRBV 20-1       N-VYGYTFGSGTRLTVV-------------------------------------------
                           ** **:*::
```

(B)

Figure S1. Sequences of TCR reactive to SMX used in Modeling Shown are the sequences obtained from varied TCC found to be reactive to SMX in previous studies. All TCC were isolated from the same patient, showing exanthema/malaise to SMX. Clone names to left correspond to the same TCC TCR in A) TCR variable α and B) TCR variable β.

Copyright © 2013 SciRes.

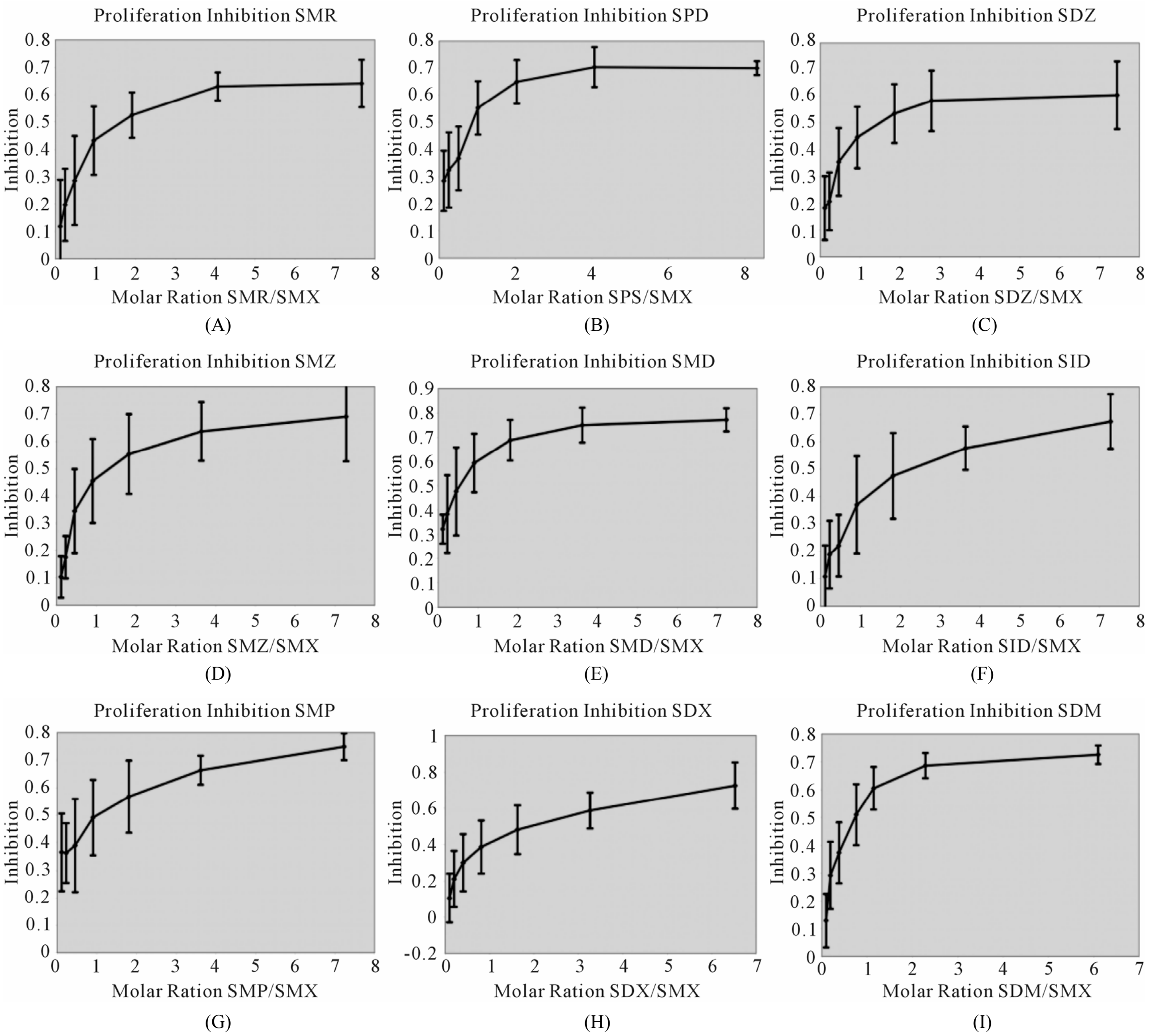

Figure S2. Proliferation Inhibition curves, other 9 SA Inhibition of SMX induced proliferation for remaining SA. Molar ratios are SA to SMX respectively. Names of SA are indicated in chart. Bars represent standard deviation, inclusive of all data from 3 triplicates each done in triplicate, representing 9 data points per tested concentration. Most compounds show maximum inhibition between a molar excess of 3 to 4 SA to SMX.

Copyright © 2013 SciRes.

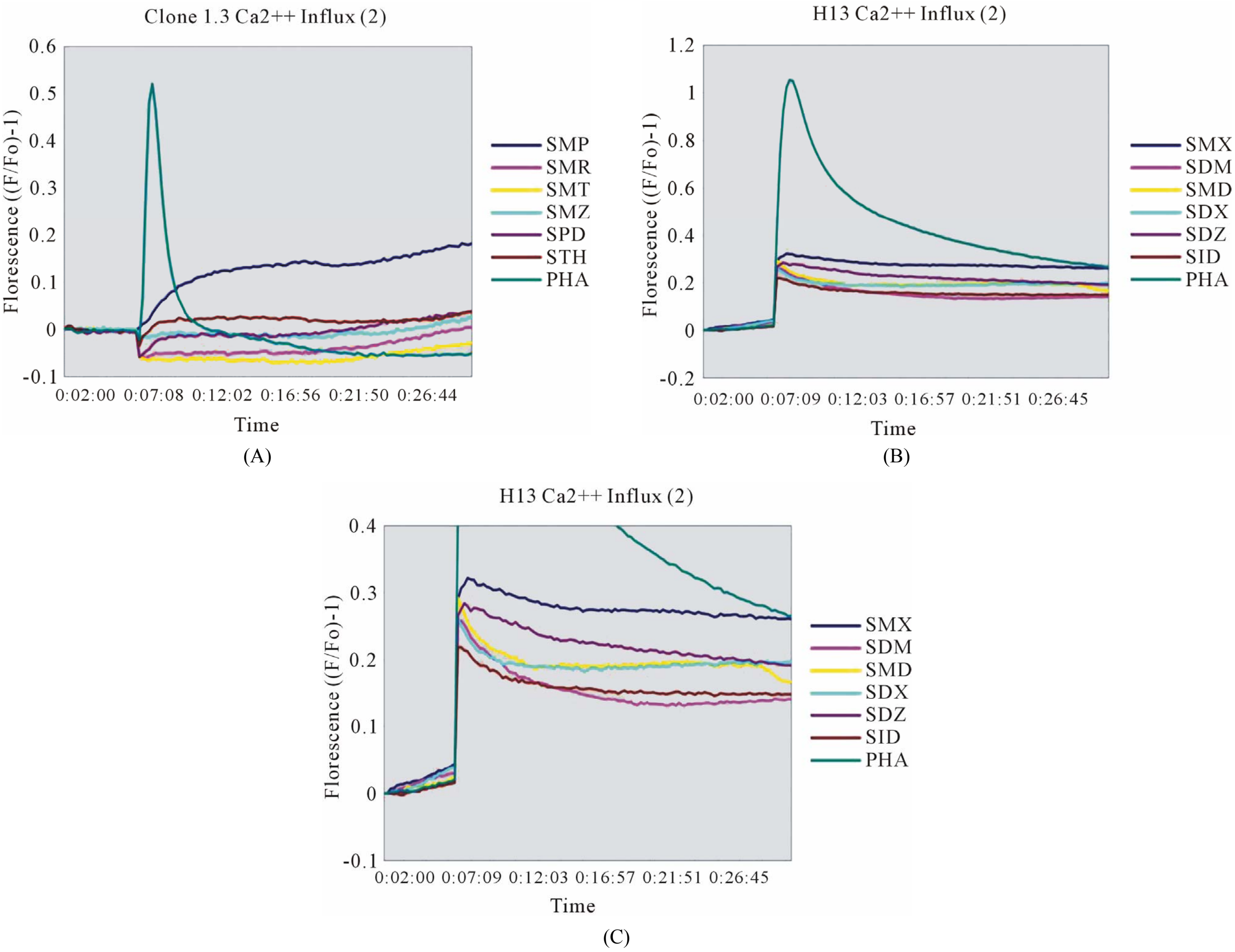

Figure S3. Other SA Ca2++ influx (A) Clone 1.3 other 6 SA tested in Ca2++. Shown is a respective SMP Ca2++ influx, with response scaled to **Figure 3** for comparison. All others gave no influx. (B) Clone H13 other 6 SA. SMX is shown as a control, only SDZ gave a response from the set of SA shown. (C) Zoom of (B) to show more precise influx scale.

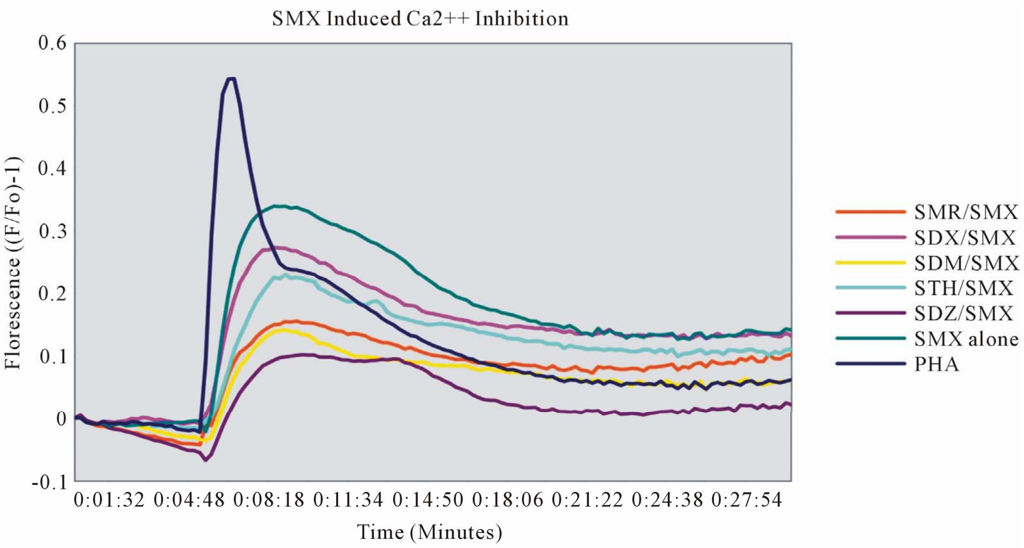

Figure S4. Ca2++ Inhibition of TCC 1.3, remaining 6 SA Inhibition of SMX induced Ca2++ influx for remaining 6 SA of TCC 1.3. All inhibitions were are conducted at a molar ratio of 1:1 SA to SMX.

Copyright © 2013 SciRes.

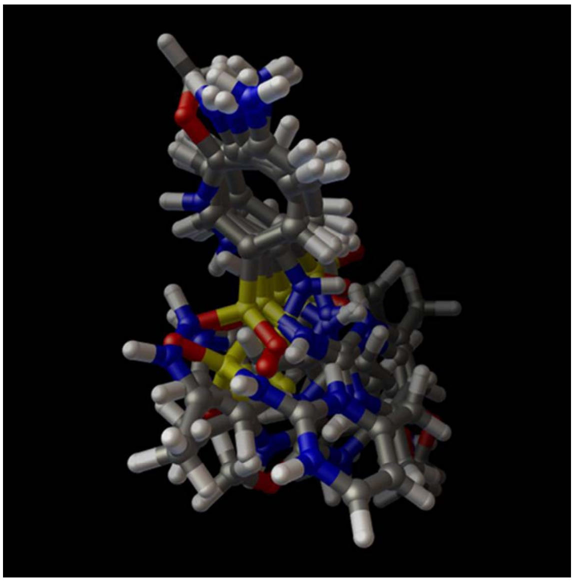

Figure S5. Overlay of all SA in CDR3α of Clone 1.3 Overlay of all SA docked into the CDR3α loop of clone 1.3. Top of figure, TCR, bottom MHC-peptide groove. Each was docked, then energy minimized until no change in energy was observed using GROMACS. The NH2 of the terminal SA core shows no movement due to the strong interaction with CDR1β TYR 31, while R groups show a wide degree of displacement. SMP is overlaid in reversed docked conformations, to illustrate orientation, where the TYR 31 is displaced by the CH3 group.

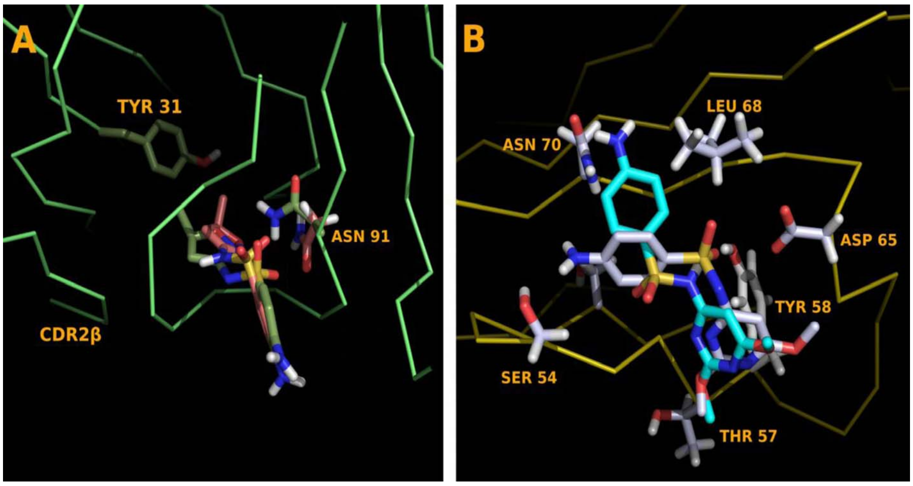

Figure S6. Dehydrated and Hydrated SA Comparisons (A) Dehydrated, green, and hydrated SMX, pink docked into the CDR3α loop of clone 1.3. The hydrogen is drawn into SMX, normally not represented in figures to show the offset caused by removal. The molecule rotates, allowing the N of the neck-R group to form a hydrogen bond with backbone H between position 95 and 96. An additional hydrogen bond deficit is made through ASN 91, which forms a bond to the displaced o of the SO2. (B) Dehydrated, gray and non-dehydrated SDM, aqua. A change in the S-N-R group angle from 110˚ to 127˚ allows the SA core to be properly placed in the binding pocket. Amino acids of the CDR2β loop of H13 are labeled.

Copyright © 2013 SciRes. OPEN ACCESS
